# A micro dataset on delinquency risk, loan motivations and credit access in Chile

**DOI:** 10.1016/j.dib.2019.104684

**Published:** 2019-10-18

**Authors:** Carlos Madeira

**Affiliations:** Central Bank of Chile, Chile

**Keywords:** Consumer credit, Default risk, Interest rate ceilings, Usury laws

## Abstract

This article provides data that allows to estimate how delinquency risk and consumer loan motivations changed in Chile before and after the new Interest Rate Ceiling Law of 2013. The data is of particular interest for those interested in the heterogeneity of household borrower profiles and their reactions to new loan regulation. The codes are in Stata format and the datasets are in Excel.

**Table:** Specifications Table

Subject	Economics, Econometrics and Finance.
Specific subject area	Household Finance. Impact evaluation of programs and legislation.
Type of data	Table (Excel format – Stata do files are included that show how to create other summary tables and figures.)
How data were acquired	Data combines publicly available raw data from the Chilean Household Finance Survey (EFH, waves 2007 until 2014) and matches it with unemployment risk from similar workers in the Chilean Employment and Income Survey (ENE/ESI). Hardware: data analysis was performed in a standard notebook with an Intel Core i7-4700HQ 2.40GHz processor with 16.0 GB of RAM. Software: Stata MP-6 (version 15.1).
Data format	Raw (CS_debts_EFHall2.xlsx and EFH_TMC_CS.xlsx) and Analysed (see the folder “analysed_data” with 12 brief Excel files detailed in Table 1 in this article).
Parameters for data collection	The data consists of national urban households in Chile, with 16,770 households interviewed at the cross-sectional level. Demographic variables (age, education, labour income) were collected at the individual level, while loans and assets were elicited at the household level.
Description of data collection	Data formats the raw survey information of the Chilean Household Finance (EFH), adding simple variables of borrower status, loan delinquency, debt motivations, permanent income and unemployment.
Data source location	Central Bank of Chile, Santiago, Chile.
Data accessibility	With the article.
Related research article	Madeira, C. (2019), “The impact of interest rate ceilings on households' credit access: Evidence from a 2013 Chilean legislation”, *Journal of Banking and Finance*, 106, 166–179. https://doi.org/10.1016/j.jbankfin.2019.06.011

**Table:** 

**Value of the Data** • The data can be used to study how delinquency risk changed before and after the 2013 Chilean Maximum Interest Rate law (see Córdova and Toro [2], Cuesta and Sepúlveda [3], Hurtado [4], and references therein). This is an important regulation topic for several countries (Maimbo and Henriquez [5]), especially as household credit keeps growing. • The data can be used by researchers interested in delinquency risk of consumer loans and how these relate to demographics (age, civil status, college education, number of household members), household income (currently monthly income measure at the time of the survey, annual permanent household income), indebtedness (the ratio of consumer debt to annual permanent household income, the ratio of total monthly debt service to household income), and unemployment risk. The data includes dummy variables for mortgage and consumer loan delinquency contracts after 30 days or more and after 90 days or more. It also contains measures of payment difficulties for retail and banking credit cards. • Researchers can use the insights of this data to study how interest rate ceilings are likely to affect developing countries, accounting for heterogeneity of families and how the impact may depend on characteristics such as age and income. Since most underdeveloped countries have scarce microeconomic data on this topic (Maimbo and Henriquez [5]), the dataset is likely to be influential with policy makers, international institutions, besides academic researchers. • Finally, the data is useful for researchers interested in studying how borrowers' behaviour and motivations differs across different lenders. In particular, the data includes information on the debtor status of households: Both Banks and Retail Stores, Banks only, Retail Stores only, Labour and Credit Unions, Other Loans, whether households have no wish for consumer debt (No Wish for Debt), or whether households applied for loans but were refused (No Access to Debt). The data also includes measures of the share of consumer loans motivated by “Current consumption purchases”, “Durable goods and investments”, “Pay previous debts”, and “Health”.

## 1 Data

Two data files are included: CS_debts_EFHall2.xlsx and EFH_TMC_CS.xlsx. These datasets can be easily imported to Stata for analysis. These are the raw micro datasets.

The data file CS_debts_EFHall2.xlsx contains 21,319 cross-sectional household observations from the Chilean Household Finance Survey (in Spanish, *Encuesta Financiera de Hogares*, hence on EFH) for the survey waves 2007, 2008, 2009, 2010, 2011, 2014 and 2017. It provides information on the households’ demographic characteristics (years of education, age, number of household members), income (monthly income, income strata at the national level, with strata given by the percentiles 1–50, 51–80, 81–100), and consumer loan amount (in a real monetary index labelled as UF, which is widely used in Chile – one UF corresponds roughly to 40 USD during the period of analysis). It also gives information on whether the household has a loan of 0–50 UF or 50–200 UF with banks or with non-banking lenders. The dataset is at the level of the household head (and does not include information on the individual family members).

The data file EFH_TMC_CS.xlsx contains data for all the households in the EFH survey between 2007 and 2014 and it includes information on all the individual family members. It has observations on 16,770 households and 56,986 household members, with information on the role of each household member (household head, spouse/partner, child, sibling, parent or in-law, other relative, or domestic worker). It has information on the household members’ age, gender, civil status, monthly income, number of household members, monthly debt service at the household level, delinquency status (1 month or more in arrears, 3 months or more in arrears) for mortgage loans, overall consumer loans, banking consumer loans, banking and retail credit cards. Finally, it contains risk-adjusted interest rate variables for consumer loans between 0-50 UF and 50–200 UF, plus dummy variables with a value of one when the risk-adjusted interest rate is above the Legal Interest Rate Ceiling in Chile for 0–50 UF and 50–200 UF, respectively.

The article also provide two Stata codes with an example of how to use these datasets: Debt_motivations_TMC.do and EFH_2011regs2.do. The code Debt_motivations_TMC.do uses the data CS_debts_EFHall2.xlsx to replicate the analysis in section 2.2 of Madeira [1]. The code EFH_2011regs2.do uses the data EFH_TMC_CS.xlsx to replicate the analysis shown in sections 2.2 and 3.2 of Madeira [1]. These 2 codes create the analysed final tables in sections 2.2 and 3.2 of Madeira [1]. The Stata codes use the raw datasets to replicate the 12 analysed data Excel files in the folder “analysed_data” described succinctly in Table 1 below.

**Table 1:** Description of the analyzed datasets provided in this article (these datasets can be replicated exactly using the 2 raw datasets – CS_debts_EFHall2.xlsx and EFH_TMC_CS.xlsx – and the 2 companion Stata codes, Debt_motivations_TMC.do and EFH_2011regs2.do).

Analyzed dataset	Description
df_dna_06_11.xls	Delinquency risk coefficients obtained from a probit model using the EFH data, with first column using the entire 2007–2014 sample, second column using the 2007–2011 sample, third one using the 2007 sample, fourth one using the 2008 to 2010 sample, and fifth one using the 2014 sample.
LS_DTyear.xls	Data provides the fraction of families that are classified in 7 borrower status categories, their loan motivations and fraction of borrowers in delinquency (1 or 3 months in arrears) for consumer loans, banking consumer loans, plus bank and retail cards. It shows the fraction of borrowers restricted by the Legal Interest Rate Ceiling in Chile according to the risk-adjusted interest rate model in Madeira [1]. Data corresponds to the survey year before (2011) and after (2014) the change in the interest rate ceiling law.
LS1_DMotives.xls and LS2_DMotives.xls	Data shows the fraction of debt according to 4 loan motives for borrowers that are either restricted or unrestricted by the Legal Interest Rate Ceiling (0–50 UF) in Chile. LS2_DMotives.xls is similar but it applies the 50–200 UF Legal Interest Rate Ceiling.
LS1_DTyear.xls and LS2_DTyear.xls	Data shows the fraction of loan motives across the categories of borrower status and Loan Ceiling (0–50 UF) restricted or unrestricted status. LS2_DTyear.xls is similar, but applies the 50–200 UF Legal Interest Rate Ceiling.
Motives_DT.xls	It gives the average consumer loan value UF and loan motivations for each borrower category over the period 2007-2011-2014.
Motives_DTyear.xls and Motives_DTyear_DebtP.xls	It gives the average consumer loan value UF and loan motivations for each combination of borrower category and each year 2007, 2011 and 2014. Second file also includes 2017.
Motives_year.xls, Motives_yearDC.xls and Motives_year_DebtP.xls	Data gives the fraction of each loan motive in the total debt of each year (2011 and 2014), plus the fraction of borrowers with consumer loans in banks and non-banks of amounts 0–50 UF and 50–200 UF. Second dataset shows the fraction of loan motives across the joint category of both year (2011 and 2014), borrower status and debt size (0–50 UF and 50–200 UF). Third dataset shows the values of loan motives for the consumer debt for each survey year (2007 until 2017).

## 2 Experimental design, materials, and methods

The data consists of a formatting of the EFH dataset, which creates delinquency variables at the household level for mortgage and consumer loans, loan motivations, and non-payment of banking or retail credit card debt. Household measures of permanent income and unemployment risk were included based on workers of similar characteristics from the Chilean Employment and Income Survey (in Spanish, Encuesta Nacional de Empleo (ENE) and Encuesta Suplementaria de Ingresos (ESI), hence on ENE/ESI, waves between 2007 and 2014) of the same quarters of the respective EFH survey interviews. The data published with this article has a similar format for all the EFH waves: 2007, 2008, 2009, 2010, 2011 and 2014. Users can apply for the raw data of all the EFH and ENE/ESI surveys by filling the respective forms on the websites of the Central Bank of Chile (http://www.bcentral.cl) and the Chilean Institute of National Statistics (http://www.ine.cl/ene/base-de-datos-ene.php).

The sequence for the final data creation can be described as follows:

1) The EFH dataset (waves 2007 to 2014) are used, which includes a total of 16,770 urban households interviewed at the cross-sectional level. Socioeconomic information on age, years of education, three income strata (national household income percentiles 1 to 50, 51 to 80, and 81 to 100), number of household members, total monthly household income, a dummy variable for the Santiago Metropolitan Region and the type of county (low income level, high income level, and upper income level) is obtained directly from the survey. Variables of delinquency in arrears and credit card non-payment are included.
2) The variable “deudacon” classifies all the EFH households into 7 mutually exclusive categories of borrowers according to their largest consumer loan amount held: 1) borrowers in Banks (but not in Retail Stores), 2) households with consumer loans both in Banks and Retail Stores, 3) households with loans in Retail Stores (but not in Banks), 4) households with consumer loans in Labour and Credit Unions, 5) Other Loans (car sellers, pawnshops, informal loans), 6) households with No Desire for Debt, and 7) households with No Access to Debt (because their loan applications were rejected or expected to be rejected).
3) The share of consumer debt represented by each of 4 types of loan motivations (“Current consumption purchases”, “Durable goods and investments”, “Pay previous debts or loan consolidation”, and “Health”) is created by grouping more detailed loan categories. In particular, the category “Current consumption purchases” is an aggregation of the motivations: “Purchase of articles for the home and living expenses”, “To purchase clothes”, “Other”. The category “Durable goods and investments” also aggregates more detailed classifications of motives: “To buy a vehicle or other means of transport, maintenance and repair expenses of vehicles”, “Vacations”, “To finance a business or professional activity”, “For investment in financial assets”, “To refurbish or renovate the residence”, “For education purposes”, “To purchase real estate assets”, “To provide funds or make a loan to another person or relative”.
4) Using data from workers of similar characteristics from the Chilean Employment Survey (ENE/ESI), conditional on their education, age, industry, income quintile and region, a measure of permanent labour income was built for each household member, P_k(i),t_, accounting for their earnings while employed, their probability of unemployment spells and their replacement ratio of earnings while in unemployment. The households’ permanent income is obtained as the sum of their non-labour income plus the permanent income of their members: P_i,t_ = a_i_+∑_k_ P_k(i),t_. The household unemployment risk is estimated as a permanent-income weighted average of the unemployment risk of its labour force members: u_i,t_ = ∑_k_ P_k(i),t_/(P_i,t_-a_i_)u_k(i),t_.

All the methods (in Stata do-files) are published in a RunMyCode companion website: http://www.runmycode.org/companion/view/3576.
